# Development of FRET Biosensor to Characterize CSK Subcellular Regulation

**DOI:** 10.3390/bios14040206

**Published:** 2024-04-20

**Authors:** Mingxing Ouyang, Yujie Xing, Shumin Zhang, Liting Li, Yan Pan, Linhong Deng

**Affiliations:** 1 Institute of Biomedical Engineering and Health Sciences, School of Medical and Health Engineering, Changzhou University, Changzhou 213164, China; xyujie1128@163.com (Y.X.); zsm@cczu.edu.cn (S.Z.); liliting190@163.com (L.L.); py@cczu.edu.cn (Y.P.); 2 School of Pharmacy, Changzhou University, Changzhou 213164, China

**Keywords:** C-terminal Src kinase (CSK), fluorescence resonance energy transfer (FRET), Src family kinase, PTPα, kinase submembrane regulation

## Abstract

C-terminal Src kinase (CSK) is the major inhibitory kinase for Src family kinases (SFKs) through the phosphorylation of their C-tail tyrosine sites, and it regulates various types of cellular activity in association with SFK function. As a cytoplasmic protein, CSK needs be recruited to the plasma membrane to regulate SFKs’ activity. The regulatory mechanism behind CSK activity and its subcellular localization remains largely unclear. In this work, we developed a genetically encoded biosensor based on fluorescence resonance energy transfer (FRET) to visualize the CSK activity in live cells. The biosensor, with an optimized substrate peptide, confirmed the crucial Arg^107^ site in the CSK SH2 domain and displayed sensitivity and specificity to CSK activity, while showing minor responses to co-transfected Src and Fyn. FRET measurements showed that CSK had a relatively mild level of kinase activity in comparison to Src and Fyn in rat airway smooth muscle cells. The biosensor tagged with different submembrane-targeting signals detected CSK activity at both non-lipid raft and lipid raft microregions, while it showed a higher FRET level at non-lipid ones. Co-transfected receptor-type protein tyrosine phosphatase alpha (PTPα) had an inhibitory effect on the CSK FRET response. The biosensor did not detect obvious changes in CSK activity between metastatic cancer cells and normal ones. In conclusion, a novel FRET biosensor was generated to monitor CSK activity and demonstrated CSK activity existing in both non-lipid and lipid raft membrane microregions, being more present at non-lipid ones.

## 1. Introduction

The discovery of Src, the first member of the Src family kinases, dates back to the 1970s, when it was identified as a transformed gene in the Rous sarcoma virus (RSV) [1,2,3]. Subsequent studies identified other relevant kinases, collectively known as SFKs, including Yes, Fyn, Lyn, and Lck [4,5]. SFKs’ activity is regulated by phosphorylation events. In the late 1980s, researchers studied the mechanism of the negative regulation of SFKs and showed that the phosphorylation of the C-tail tyrosine site inhibited SFKs’ activity. They focused on identifying the kinases responsible for the phosphorylation of inhibitory tyrosine residues. Through biochemical and genetic methods, they identified and characterized C-terminal Src kinase (CSK) as the kinase responsible for this inhibitory phosphorylation [6,7].

The CSK molecule weighs 50 kDa and consists of three functional domains: the SH3, SH2, and kinase domains [8]. In contrast to SFKs’ structures [9], there is no N-terminal acylation group in CSK, whose regulatory tyrosine is located in the kinase and C-terminal domains [10]. Without a transmembrane domain or fatty acyl modifications, CSK is mainly present in the cytoplasm, while its substrate SFKs are immobilized on the membrane by the myristic and palmitate moieties of the N-terminus. Therefore, the translocation of CSK to cell membranes that inactivate SFKs is considered a critical step in CSK regulation [11]. While the transfer of CSK to the cell membrane requires binding to its membrane anchor, Masato Okada et al. successfully isolated a transmembrane adapter-like molecule, CBP/PAG1 (CSK-binding protein/phosphoprotein associated with a glycophospholipid-rich membrane), from a cholesterol-rich membrane microdomain or “lipid raft” [12,13]. Recently, it has been shown that when CBP is phosphorylated by active SFKs, CSK is recruited to the lipid raft by binding to CBP, and CSK phosphorylates the C-terminal Y527 of SFKs, resulting in an intramolecular interaction between the phosphorylated tyrosine and the SH2 domain of SFKs [14,15]. This interaction places the SFKs in an inactive conformation and prevents their activation [16]. Inactivated SFKs can be activated by PTPα dephosphorylation [17]. CSK plays a pivotal role in numerous cellular processes, including apoptosis, cell proliferation, cancer invasion, and angiogenesis, primarily through the regulation of SFKs; furthermore, it contributes to the inhibition of cancer progression, as SFKs have been linked to the development of various cancers [16].

Receptor-type protein tyrosine phosphatase alpha (PTPα) is a 130 kDa transmembrane phosphatase with wide expression [18]. In 1992, Zheng et al. established a rat embryo fibroblast (REF) model overexpressing PTPα and found that the overexpression of PTPα could specifically catalyze the dephosphorylation of the tyrosine residue at position 527 (corresponding to human c-Src 530) of the c-Src C-terminus, which continuously activated C-Src and induced the malignant transformation of cells [19]. With the deepening of the research, Zheng et al. further proposed a “displacement model”: PTPα pTyr789 binds to Grb2 SH2-c-SH3 at rest, while c-Src pTyr527 preferentially binds to self-SH2 [20]. Under certain activation signals, PTPα pTyr789 dissociates from Grb2 and in turn binds to c-Src SH2 and dephosphorylates pTyr527, thereby activating c-Src. The protein tyrosine kinase CSK can phosphorylate the 527-position tyrosine at the C-terminal of Src, so we further explored the relationship between these two different proteins acting on the same site of Src.

Although membrane localization is important in the regulation of CSK’s function, there is a lack of imaging tools that can directly visualize CSK’s activity at the plasma membrane and its submembrane microdomains. Fluorescence resonance energy transfer (FRET) technology and genetically encoded FRET biosensors provide powerful tools for the monitoring of biochemical activity and visualization of signaling molecules in vitro and in living cells [21,22,23]. The FRET donor and acceptor pair, consisting of enhanced cyan fluorescent protein (ECFP) and a variant of yellow fluorescent protein (YPet), has been optimized for FRET biosensor engineering; it can form weak dimers that enhance the efficiency and sensitivity of FRET [24]. This FRET pair has also been used to design a range of highly sensitive biosensors, such as the Rac1, Syk, and ZAP70 FRET [25].

While the choice of donor and acceptor fluorescent proteins has a great influence on the sensitivity of the biosensor, the SH2 domain and substrate tyrosine peptide are key factors in determining the specificity and sensitivity of the biosensor. Therefore, using the ECFP/YPet FRET pair, we designed the CSK FRET biosensor and characterized the sensitivity and specificity in ASM cells to select the optimal biosensor. The activity of CSK is detected by the FRET reactions of the biosensors in cancer cells. By localizing the biosensor to the membrane microregions of ASM cells, we observed that the non-lipid raft region has a large amount of active CSK. Thus, our work provides a CSK FRET biosensor with relative sensitivity and specificity for live cell imaging and further reveals the mechanism by which different membrane spacings in ASM cells regulate CSK activity.

## 2. Materials and Methods

### 2.1. DNA Constructs

The construct of the CSK biosensor was first subcloned using the vector pRSETb for bacterial expression, according to the procedures described previously [25]. The DNA fragment containing the sequence of substrate peptide “FTSTEPQYQPGENL” from the Src tail was amplified by PCR using the Fyn biosensor as the template [25]. The biosensor in pRSETb was further subcloned into the vector pCAGGS for mammalian expression [26]. Two other substrate versions (FTATEPQYQPGENL and EEEIYFFF) of the biosensor and the Y/F mutant in the substrates were constructed in the same way. To generate the membrane-targeted biosensors, the Lyn tag with 21aa (MGCIKSKRKDNLNDDGVDMKT) or the Fyn tag (MGCVQCKDKEATKLTEERDGSLNQ) was added at the N-terminus of the biosensor [25]. The KRas tag (KKKKKKKSKTKCVI) [27] was added to the C-terminal of the biosensor in the same way.

### 2.2. Reagents and Cell Culture

ASM cells were maintained in low-glucose Dulbecco’s modified Eagle’s medium (DMEM, Sigma-Aldrich, St. Louis, MO, USA) supplemented with 10% fetal bovine serum (FBS, Thermo Fisher Scientific, Waltham, MA, USA), 100 μg/mL penicillin, and 100 unit/mL streptomycin at 37 °C with 5% CO_2_ in a humidified incubator. The MDA-MB-231 cells were cultured in RPMI-1640 (Thermo Fisher Scientific, Waltham, MA, USA). The MCF-10A cells were cultured in advanced DMEM/F12 medium supplemented with 20 ng/mL epidermal growth factor (EGF), 10 μg/mL insulin, 10 ng/mL cholera toxin, and 250 ng/μL hydrocortisone. BEAS-2B cells were maintained in high-glucose DMEM (Thermo Fisher Scientific, Waltham, MA, USA), and A549 cells in Ham’s F12 nutrient mixture (Thermo Fisher Scientific, Waltham, MA, USA). The culture medium was supplemented with 10% fetal bovine serum (FBS, Thermo Fisher Scientific, Waltham, MA, USA), penicillin (100 unit/mL), and streptomycin (100 ng/mL). The cells were cultured at 37 °C in a humidified incubator with 5% CO_2_.

Fibronectin was purchased from Thermo Fisher Scientific. Rat platelet-derived growth factor (PDGF-BB) and human epidermal growth factor (EGF) were purchased from Sigma.

### 2.3. Cell Transfection with DNA

The DNA constructs were transfected into ASM cells with Lipofectamine 3000 reagent (Invitrogen, Waltham, MA, USA) 2–3 days before the imaging experiments. Generally, 1 μg biosensor DNA was transfected into cells per well in a 24-well plate. For the comparison of the FRET responses to different kinases in the cells, 1 μg biosensor construct was co-transfected with plasmids encoding the different kinases (in the amount indicated later). During co-transfection, the different DNA plasmids were mixed thoroughly by pipetting them up and down 10–20 times in an Eppendorf tube (Eppendorf, Hamburg, Germany) and then mixed with Lipofectamine 3000 reagent at a ratio of 1:2 (μg DNA:μL Lipofectamine). After transfection for 8–16 h, cells were maintained in low-serum DMEM containing 0.5% FBS and then seeded on 10 μg/mL fibronectin-coated glass-bottom dishes (NEST, Palo Alto, CA, USA) in low-serum DMEM for about 24 h before FRET imaging.

### 2.4. Microscope and Image Acquisition

The FRET imaging and quantification procedures followed similar protocols to those described in our recent publications [25,28]. In summary, we employed a Zeiss microscopy system (Zeiss Cell Observer, Jena, Germany) equipped with multi-position capabilities, precise auto-focusing, and an automatic-switchable dichroic rotator. To maintain a constant temperature of 37 °C and 5% CO_2_ for live cell samples, the microscope stage was supplemented with a Zeiss incubator box. The parameters of the excitation filter and dichroic mirror for FRET image acquisition were 436 ± 10 and 455 nm, and the emission filters were 475 ± 20 nm and 535 ± 15 nm for the ECFP and FRET (YPet) channels, respectively. Twenty to thirty positions from each sample dish were selected and imaged under the same condition. For PDGF or EGF stimulation during FRET imaging, 1 mL DMEM containing the growth factor was injected into the sample dish by a syringe guided through a microtube, which did not disrupt the imaging process.

### 2.5. FRET Quantification

FRET assessments were carried out using FluoCell, a MATLAB tool developed by the Wang Lab at UCSD [29]. Following background subtraction, fluorescence signals from ECFP and FRET (YPet) images underwent pixel-by-pixel calibration. GraphPad Prism 6 and Excel 2016 were employed for data processing and statistical analysis. Quantified FRET data were represented as time-course curves (mean ± S.E.M.) and scatter plots (mean ± S.D.), with significance levels marked by *, **, ***, and ****, indicating *p*-values < 0.05, 0.01, 0.001, and 0.0001, respectively, using Student’s *t*-test. Multiple *t*-tests were conducted between the control and experimental groups under various conditions. All FRET experiments were independently replicated on different days, with statistical analyses based on data collected at different time points.

## 3. Results

### 3.1. Biosensor Design and Characterization of Sensitivity and Specificity of CSK Biosensor in Mammalian Cells

**Figure 1:**
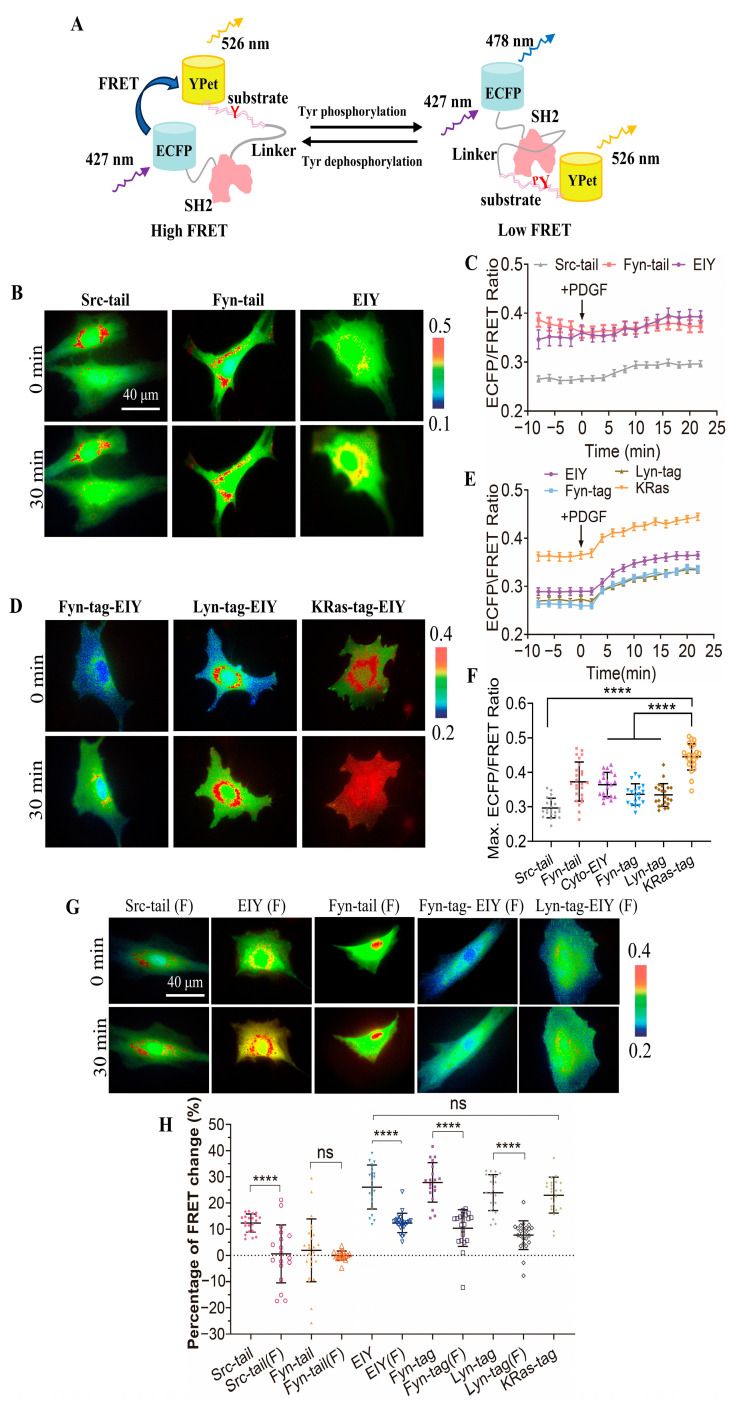
Characterization of the designed CSK biosensors with different substrate peptides and submembrane localization. (**A**) A schematic illustration representing the structural alteration of the biosensor due to tyrosine phosphorylation on the substrate. (**B**,**C**) ECFP/FRET ratiometric images of ASM cells treated with 10 μg/mL PDGF. The multiple versions of the biosensor consisted of different substrate peptides (**B**) or submembrane-targeting signal peptides (**C**). The gradient color scale ranging from blue to red indicates the emission ratio (ECFP/FRET) of the biosensor, reflecting the transition from low to high values. (**D**,**E**) The temporal trends of the ECFP/FRET emission ratio measured for the cells in (**B**,**C**). (**F**) The scatter plots (mean ± S.D.) compare the basal and maximal levels of the ECFP/FRET ratio in cells, corresponding to the time courses depicted in (**D**,**E**). (**** indicates *p* < 1.0 × 10^−4^, *n* = 21, 25, 20, 19, 22, 23). In cells transfected with Src-tail, Fyn-tail, and EIY CSK-FRET (1.0 μg of DNA on a 24-well plate), the ECFP/FRET ratio values (mean ± S.D.) were 0.297 ± 0.028, 0.373 ± 0.057, and 0.36 ± 0.035 at the peak after PDGF stimulation, respectively. For Fyn-tag, Lyn-tag, and KRas-tag CSK-FRET, the ratio values were 0.33 ± 0.031, 0.33 ± 0.033, and 0.44 ± 0.035 at the peak, respectively. (**G**) ECFP/FRET ratiometric images of the Y/F mutant biosensors before and after treatment with 10 μg/mL PDGF. The multiple versions of the biosensor consisted of the Y/F mutant in different substrate peptides or in the EIY biosensor with submembrane-targeted peptides. (**H**) The scatter plots show the quantified FRET changes of the different CSK biosensors along with the negative Y/F mutants in response to 10 μg/mL PDGF stimulation. (**** indicates *p* < 1.0 × 10^−4^, while ‘ns’ indicates no significant difference).

In this study, we developed a FRET biosensor to monitor CSK activity in live cells. The design strategy of this biosensor is similar to that of the previously reported Fyn FRET biosensor [24,25]. Essentially, the biosensor protein consists of ECFP, the SH2 domain, a flexible linker, a specific substrate peptide for CSK, and YPet from the N-terminal to C-terminal (Figure 1A). We designed three different substrate peptides for the CSK biosensor: (1) from the Src C-terminal tail (FTSTEPQYQPGENL) [30]; (2) from the Fyn C-terminal tail (FTATEPQYQPGENL); and (3) the optimal peptide substrate for CSK determined by screening a random peptide library (EEEIYFFF) [31].

As shown in Figure 1A, when the substrate is unphosphorylated, due to the three-dimensional structure of the SH2 domain [32], ECFP is spatially close to YPet with high FRET efficiency. The tyrosine-phosphorylated substrate peptide by the kinase binds to the SH2 pocket at the opposite site, resulting in the separation of YPet and ECFP and a decrease in FRET efficiency. The FRET changes are reversible when the substrate is dephosphorylated. Therefore, the FRET efficiency of this biosensor can indicate the activity change of the CSK kinase.

The purified biosensor proteins expressed in *E. coli* showed a predicted molecular size of around 70 kDa (Figure S1 in Supplementary Materials), indicating the appropriate folding of the recombinant biosensor, consisting of multiple fragments. We characterized the biosensor containing the three different substrate peptides in mammalian cells. As the tyrosine kinase activation and the subsequent substrate phosphorylation are relatively quick responses [33], we monitored the FRET response of the biosensor within 30 min of activation by PDGF (Figure 1B). Biosensors for three different substrate peptides were separately transfected into rat airway smooth muscle (ASM) cells, and the changes in the ECFP/FRET emission ratio represented the activity of the CSK kinase. The experimental results suggest that the activity of the CSK kinase is relatively low in the cytoplasm. Interestingly, the biosensor with the substrate peptide obtained by screening a random peptide library (EEEIYFFF) showed an increased ECFP/FRET emission ratio change of approximately 26%, while the other two biosensors showed minimal changes (Figure 1D). Similar results were achieved for the three substrate-based biosensors in HeLa cells with EGF stimulation (Figure S2A,B). Therefore, the EIY version was chosen for subsequent research in this work.

### 3.2. Detection of CSK Activity with FRET Biosensor at Membrane Microregions

According to reports, CSK is a cytoplasmic protein because it does not contain acylation sites, so translocation to the plasma membrane is required to exert its inhibitory function [34,35]. Therefore, adapter proteins are required. CSK binding protein (CBP) is a transmembrane protein localized in lipid rafts [36], also known as a lipid-associated phosphoprotein. In T cells, CBP binds CSK via the SH2 domain to inhibit SFKs, and the dephosphorylation of CBP leads to the dissociation of CSK and the activation of SFK kinases [37].

To observe the activity of CSK in the detergent-resistant membrane (DRM) region, Fyn-tagged CSK and Lyn-tagged CSK biosensors were constructed by fusing a DRM-targeting motif containing myristoylation and palmitoylation sites (glycine and cysteine) derived from Fyn and Lyn, respectively, at the N-terminus of the “EEEIYFFF” substrate-based cytosolic CSK (Cyto-CSK) biosensor [38]. Additionally, a polybasic motif (Arginine) adapted from K-Ras was added at the end of the Cyto-CSK biosensor to generate the KRas-tagged biosensor, enabling its localization to the general membrane region outside the detergent-resistant membrane (DRM) [38]. Similar results have confirmed the proper localization of proteins modified by Fyn, Lyn, and KRas [38,39]. Confocal images showed membrane localization for the three versions of the biosensor (Figure S3). In line with previous studies, the Fyn, Lyn, and KRas-tagged CSK biosensors can be targeted to different microdomains on the plasma membrane successfully through distinct lipid modifications [27].

We transfected the three types of membrane-targeted biosensors and the selected EIY-cytosolic biosensor into ASM cells (Figure 1C). Upon stimulation with PDGF, all four types of biosensors showed an immediate increase in the ECFP/FRET ratio (Figure 1E, Movies S1–S4). The ECFP/FRET ratio of the KRas-CSK biosensor was significantly higher than that reported for the other two membrane-targeted biosensors and the cytosolic one, when the FRET ratio of each group of cells was measured before and after PDGF treatment (0, 20 min) (Figure 1F). As a control, the cells expressing the KRas-CSK biosensor did not show a FRET change when examined without PDGF stimulation during the same imaging process (Figure S4). The cytosolic biosensor can diffuse three-dimensionally throughout the entire cell, while biosensors localized to specific cell membrane regions are restricted to 2D membrane microdomains [25]. The differences in the local topology of the biosensors may contribute to their response dynamics. This indicates that the CSK kinase activity is primarily localized to non-DRM regions. Therefore, the KRas-CSK version was selected as the optimal CSK biosensor and is referred to as the CSK biosensor in the remaining parts of this paper.

Compared with wild-type CSK-FRET biosensors, mutant biosensors with monotyrosine (Y) mutated to non-phosphorylated phenylalanine (F) in their polypeptide substrate showed a significantly lower increase in the ECFP/FRET ratio under PDGF stimulation (Figure 1G,H). The KRas-tagged Y/F mutant was not yet generated successfully. This result demonstrates that the tyrosine phosphorylation in the designed substrate sequence corresponded to the FRET changes of the biosensor (Figure 1A) and that the FRET response of the CSK biosensor was primarily attributable to intracellular CSK kinase activity. These results further demonstrate the specificity of the designed biosensor to the CSK kinase.

### 3.3. Demonstration of SH2 Domain Crucial for CSK Activation

**Figure 2:**
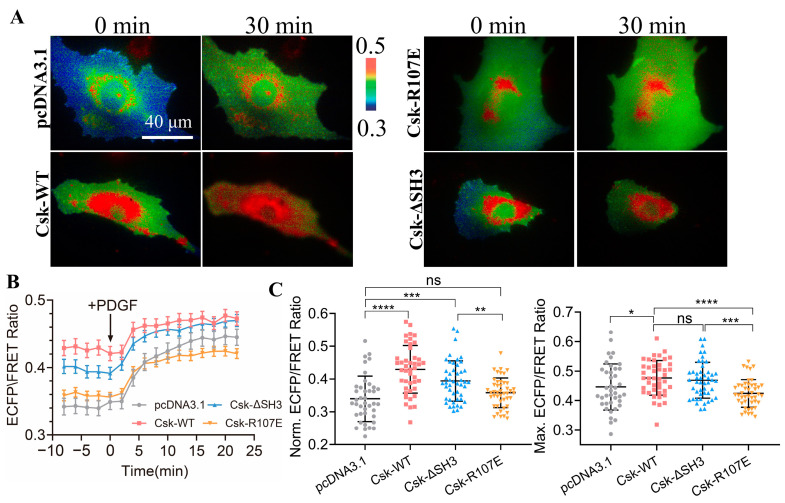
FRET changes of the biosensor in response to co-transfection of CSK constructs. (**A**) Representative ECFP/FRET ratiometric images of PDGF-stimulated ASM cells co-transfected with KRas-CSK biosensor (1.0 μg DNA per well in 24-well plate) and the vector only or the indicated CSK constructs (1.0 μg DNA each). (**B**) The time courses of the ECFP/FRET ratio in cells co-expressing the CSK mutants or control vector in (**A**). (**C**) The scatter plots compare the basal level and maximal ECFP/FRET ratio in cells with the time courses shown in (**B**). The sample sizes *n* = 40, 43, 47, 45. In cells co-transfected with the control vector, CSK (WT), CSK (ΔSH3), or CSK (R107E), the ECFP/FRET ratio values (mean ± S.D.) were 0.34 ± 0.07, 0.43 ± 0.073, 0.39 ± 0.062, and 0.36 ± 0.046 at the basal level and 0.44 ± 0.078, 0.48 ± 0.059, 0.47 ± 0.061, and 0.42 ± 0.047 at the peak after PDGF stimulation, respectively. *, **, ***, and **** indicate *p* values < 0.05, 0.01, 0.001, and 0.0001 to denote significant differences, while ‘ns’ indicates no significant difference.

In order to further validate the specificity of the CSK biosensor, plasmids of wild-type CSK (WT), SH2-inactive CSK (R107E), and CSK lacking the SH3 domain (ΔSH3) [40] were co-transfected into ASM cells. The activity of CSK was detected using the CSK-FRET biosensor, which was co-transfected with the CSK plasmids into ASM cells. PDGF was used to rapidly activate CSK in the cells. The results showed that after PDGF stimulation, the ECFP/FRET ratio in the cells immediately increased (Figure 2A,B). By analysis of the effects of different CSK plasmids on CSK activity before and after PDGF treatment, CSK (WT) and CSK (ΔSH3) led to an increase in the activity in the cells both at the basal level and upon activation, whereas the SH2-inactive CSK (R107E) did not have this effect (Figure 2C). These experiments demonstrate that the CSK-FRET biosensor has good specificity for the CSK kinase.

Yaqub et al. [41] suggested that the SH3 domain is crucial in maintaining CSK activity, whereas this conclusion has not been validated in this study. Furthermore, this experiment proves the essential role of the SH2 domain for CSK activation, which cannot be replaced or lacking.

### 3.4. Characterization of Specificity of CSK Biosensor to CSK Kinase

**Figure 3:**
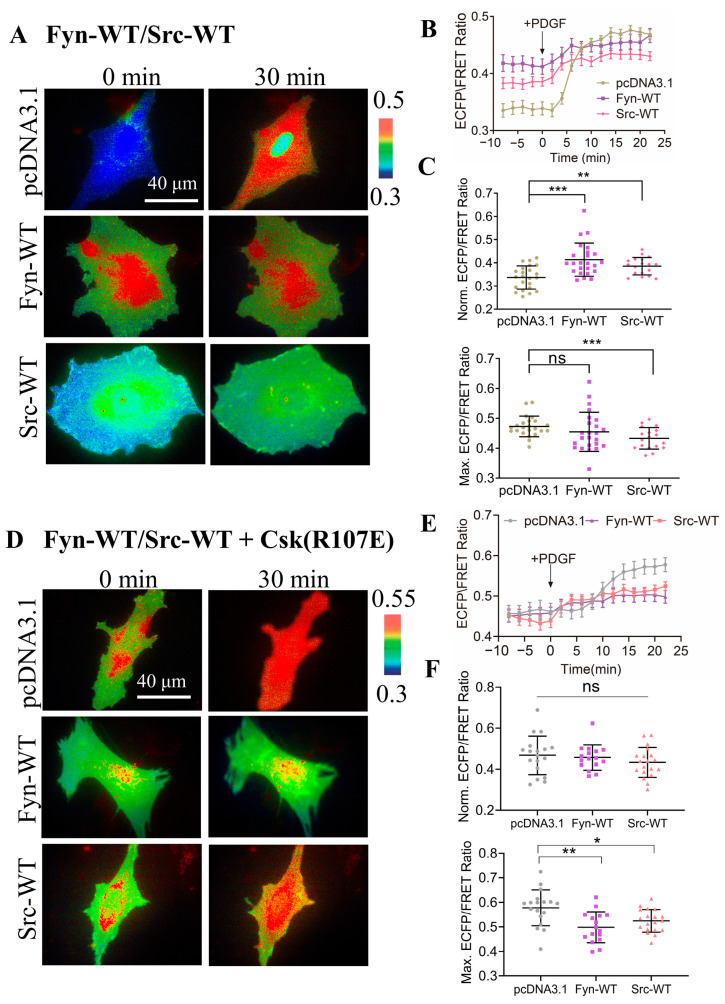
Specificity of CSK biosensor for CSK kinase. (**A**) Representative ECFP/FRET ratio images of PDGF-stimulated ASM cells co-transfected with the KRas-CSK biosensor (1.0 μg DNA in 24-well plate) with the vector only, Fyn-WT, or Src-WT (0.3 μg DNA each). (**B**,**C**) The average time courses of the ECFP/FRET ratio (mean ± S.E.M.) (**B**) and the scatter plots (mean ± S.D.) of the basal and maximal ratios (**C**) in cells co-expressing Fyn-WT, Src-WT, or the control vector in (**A**) treated with PDGF. The sample sizes *n* = 23, 24, 18. (**D**) Representative ECFP/FRET ratio images of PDGF-stimulated ASM cells co-transfected with the KRas-CSK biosensor (1.0 μg DNA in 24-well plate) and CSK-R107E (0.5 μg DNA each) with the vector only, Fyn-WT, or Src-WT (0.5 μg DNA each). (**E**,**F**) The time courses of the ECFP/FRET ratio (**E**) and the scatter plots (mean ± S.D.) (**F**) in cells (**D**) treated with PDGF. *n* = 18, 16, 19. *, **, and *** indicate *p* values < 0.05, 0.01, and 0.001 to denote significant differences, while ‘ns’ indicates no significant difference.

To further examine the specificity of the CSK biosensor in mammalian cells, we introduced CSK biosensors into ASM cells along with wild-type Src (Src-WT) and wild-type Fyn (Fyn-WT) and measured the ECFP/YPet emission ratio (Figure 3A). The time curves of the quantitative analysis indicate that the CSK activity was effectively activated under both Src WT and Fyn WT expression conditions (Figure 3B). Further statistical comparison showed that the CSK activity was significantly higher in cells expressing Src-WT and Fyn-WT than in the control group before PDGF stimulation. It is speculated that CSK is a negative regulator of SKFs, and the overexpression of Src kinase and Fyn kinase in cells promotes the activation of the CSK kinase. However, after PDGF stimulation, there was no significant difference between the experimental group expressing Fyn-WT and the control group. Interestingly, the CSK activity was reduced in the experimental group expressing Src-WT, which needs further study (Figure 3C).

In considering cells expressing endogenous CSK, the same co-transfection method was used to simultaneously transfect inhibitory CSK (R107E) on the basis of the above experiments (Figure 3D). After PDGF stimulation, the time curve of the quantitative analysis indicated that the CSK activity could be effectively activated under the different conditions (Figure 3E). Further statistical comparison of the differences showed that, before PDGF stimulation, there was no significant difference between the experimental group and the control group (pcDNA3.1), while, after PDGF stimulation, the intracellular CSK activity was significantly inhibited in the experimental group (Figure 3F). These results suggest that, in mammalian cells, the substrate tyrosine is mostly phosphorylated by CSK, leading to changes in the FRET of the biosensor.

### 3.5. Comparison of Activity Levels of Different Kinases in ASM Cells

**Figure 4:**
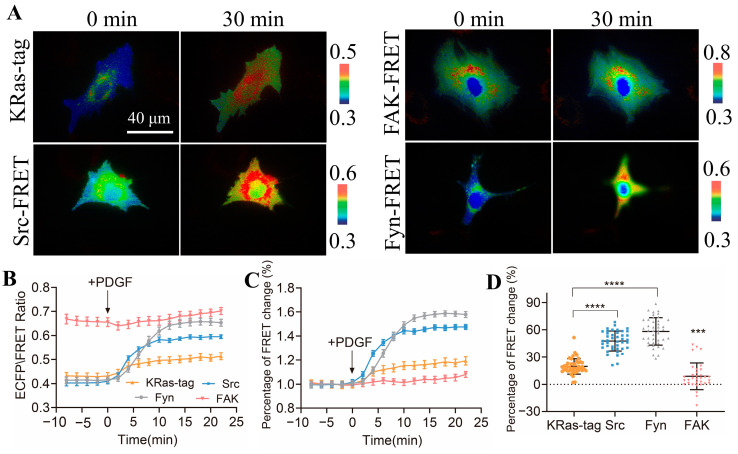
Comparison of PDGF-induced FRET responses among multiple biosensors in ASM cells. (**A**) Representative ECFP/FRET ratio images of PDGF-stimulated ASM cells transfected with CSK-FRET, Src-FRET, Fyn-FRET, and FAK-FRET biosensors with plasma membrane localization. (**B**) The measured temporal profiles of the ECFP/FRET emission ratio for the cells depicted in (**A**). (**C**) The normalized time courses of the FRET changes in (**A**). (**D**) The scatter plots show the quantified FRET changes of the biosensor in response to 10 μg/mL PDGF. The sample sizes *n* = 43, 42, 42, 33. ***, and **** indicate *p* values < 0.001, and 0.0001 to denote significant differences.

Biosensors based on FRET technology have been developed to detect the activity of Src, Fyn, and focal adhesion kinase (FAK), which are very mature biosensors with verified specificity and sensitivity. We compared the FRET responses of the constructed CSK biosensor with those of these three biosensors to study the activity differences in these kinases in the membrane microregions of the cells. The four biosensor plasmids were transfected in ASM cells, including KRas-CSK FRET, FAK-FRET [27], Src-FRET [42], and Fyn-FRET [25], respectively, and the FRET responses were stimulated with PDGF in the cells (Figure 4A,B). The quantification data showed that the ECFP/FRET emission ratio change of KRas-CSK-FRET was 19%, that of FAK-FRET was 3%, that of Src-FRET was 44%, and that of Fyn-FRET was 60% (Figure 4C,D). This indicates that in the membrane microregions, the activity of the CSK kinase is lower than that of the Fyn kinase and Src kinase, but higher than that of the FAK kinase. Hence, the activity of the CSK kinase in the membrane microregion is relatively mild in comparison to the high activity levels of the Src and Fyn kinases.

### 3.6. Protein Tyrosine Phosphatase PTPα in Regulating CSK Kinase Activity

PTPα can activate c-Src and other Src family kinases (SFKs) [18,43]. Fibroblasts from PTPα gene knockout mice exhibited enhanced Src phosphorylation at Tyr527, resulting in decreased Src activity [43,44]. Previous studies have demonstrated that PTPα can regula te the invasiveness of colon cancer cells, as confirmed by in vitro experiments using the chicken chorioallantois membrane assay [45].

When investigating the mechanism by which PTPα regulates Src activity, researchers found that the Tyr789 residue of PTPα is essential for the binding of PTPα to Src and for Src dephosphorylation and activation. Tyr789 of PTPα binds to the Src SH2 domain [46,47], leading to the exposure of the phosphorylated Src carboxyl terminus and subsequent dephosphorylation, which activates Src [48]. PTPα is compositionally phosphorylated on Ser180, Ser204, and Tyr789 [49]. In the interphase, the phosphorylation of Ser204 inhibits the binding of Src, so Src is not activated. In mitosis, pSer204 dephosphorylation leads to the activation of PP2A. Non-phosphorylated Ser204 binds to Src via regions that have not yet been identified in Src, and PTPα, while maintaining phosphorylation on Tyr789, binds to the adaptor protein GRB2 [50].

**Figure 5:**
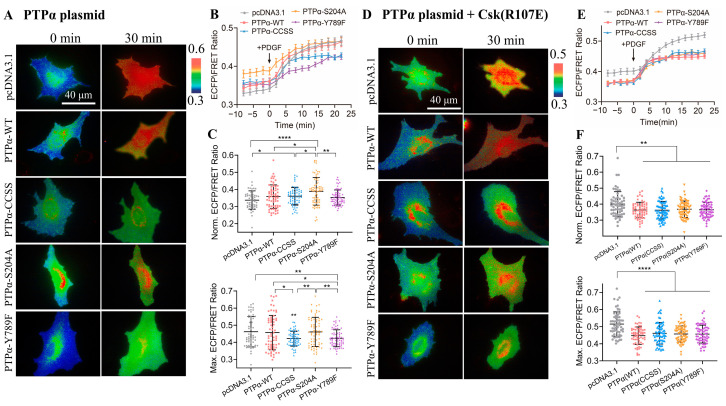
The regulation of CSK FRET responses by co-transfected PTPα constructs. (**A**,**D**) Representative ECFP/FRET ratio images of PDGF-stimulated ASM cells co-transfected with the KRas-CSK biosensor (1.0 μg DNA each in 24-well plate) with the vector only or the indicated PTPα mutants (1.0 μg DNA each) (**A**), or co-transfected with the KRas-CSK biosensor (1.0 μg DNA each) and CSK-R107E (0.5 μg DNA each) with the vector only or the indicated PTPα mutants (0.5 μg DNA each) (**D**). (**B**,**E**) The time courses of the ECFP/FRET ratio (mean ± SEM) in cells co-expressing the PTPα or control vector in (**A**,**D**) when they were treated with PDGF. (**C**,**F**) The scatter plots (mean ± S.D.) show the comparison of the basal and maximal ratios from the time courses in (**B**,**E**). The sample size n = 55, 61, 66, 68, 67 in (**C**); n = 58, 62,68, 66, 64 in (**F**). *, **, and **** indicate *p* values < 0.05, 0.01, and 0.0001 to denote significant differences.

The primary function of PTPα is to serve as a positive regulator of SFKs, while CSK acts as a negative regulator of SFKs. Therefore, this study examined the effect of PTPα on CSK kinase activity. Various PTPα plasmids with functional site mutations were used: wild-type PTPα(WT), PTPα with mutations in the catalytic active site (CCSS) and PTPα(S204A), and PTPα lacking key tyrosine phosphorylation sites in the C-terminus (Y789F). The time course of the quantified analysis reflected the effective activation of CSK activity under different conditions of PTPα expression (Figure 5A,B). Furthermore, the statistical comparison of the FRET levels between the experimental and control groups (pcDNA3.1) revealed that all four PTPα plasmids had a certain upregulating effect on CSK activity before PDGF stimulation, with PTPα(S204A) showing the most significant effect (Figure 5C). However, after PDGF stimulation, PTPα(CCSS) and PTPα(Y789F) exerted a certain inhibitory effect on the CSK activity, while the inhibitory effect of wild-type PTPα(WT) and PTPα(S204A), lacking the catalytic domain for phosphatase activity, was not significant (Figure 5C). Therefore, these results suggest that the expression of PTPα in cells has a certain inhibitory effect on CSK kinase activity upon PDGF stimulation.

Furthermore, this study investigated whether the inhibitory effect of PTPα acted directly on the FRET biosensor or inhibited the intracellular CSK protein. Using the same co-transfection method as described above, CSK (R107E) was co-transfected, acting as a CSK protein inhibitor. The time course of the quantified analysis again reflected the effective activation of CSK activity under different conditions of PTPα expression (Figure 5D,E). Moreover, a statistical comparison of the ECFP/FRET between the experimental groups and control (pcDNA3.1) revealed that all four PTPα plasmids exerted a certain inhibitory effect on CSK activity (Figure 5F), regardless of whether it was before or after PDGF stimulation. Therefore, this further confirms that PTPα has a certain inhibitory effect on CSK FRET responses.

In conclusion, the experimental results mentioned above demonstrate that PTPα has a certain inhibitory effect on CSK kinase activity, whether it acts on the FRET biosensor or inhibits the intracellular CSK protein. These findings provide new insights into the regulatory relationship between PTPα and CSK and offer important clues for further investigation into this mechanism.

### 3.7. Detection of CSK Activation in Cancer Cells

Since SFKs are involved in the signaling cascade of cell survival, proliferation, and migration, it is not surprising that abnormal SFK activity promotes tumorigenesis. In fact, SFK has been reported to have high levels and activity in different types of tumors, including lung, skin, colon, and breast cancer [51]. This also hints at the possible anti-tumor function of CSK. Reportedly, the CSK level was reduced in human hepatocellular carcinoma, suggesting that CSK has this antitumor effect [52]. CSK is effective in inhibiting cancer progression, while its downregulation supports tumorigenesis. The dysfunction of CSK in normal cells promotes the development of cancer [16].

**Figure 6:**
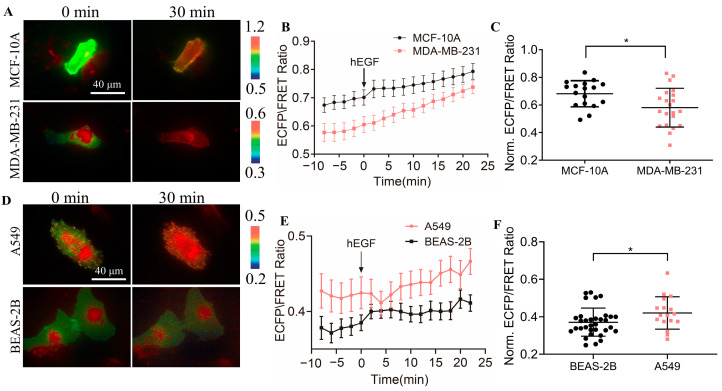
Detection of CSK FRET levels in tumor cells. (**A**,**D**) Representative ECFP/FRET ratio images of EGF-stimulated cells transfected with the KRas-CSK biosensor (1.0 μg DNA per well in 24-well plate). (**B**,**E**) The time courses of the ECFP/FRET ratio (mean ± S.E.M.) in cells when they were treated with EGF. (**C**,**F**) The scatter plots (mean ± S.D.) show the comparison of the basal ratio in cells with the time courses shown in (**B**) and (**E**), respectively. * indicate *p* values < 0.05 to denote significant differences.

Therefore, we used the designed biosensor to detect the activity of the CSK protein in normal cells and cancer cells and performed a comparative analysis. As shown in Figure 6A, the KRas-CSK biosensors were transfected in MCF-10A breast cells and malignant MDA-MB-231 breast cancer cells, and we found that the ECFP/FRET ratio in these two cell types was within 0.55~0.7 (Figure 6B). After stimulation with EGF, there was a significant difference in the activity of the CSK protein in the two cell types; in normal breast cells, the activity of the CSK protein was higher (Figure 6C). When the KRas-CSK biosensors were transfected in human normal BEAS-2B lung epithelial cells and human A549 non-small cell lung cancer cells (Figure 6D), we found that the ECFP/FRET ratio in these two cell types was within 0.35~0.5 (Figure 6E). After stimulation with EGF, the CSK protein’s activity was also different, but the activity of the CSK protein in A549 cancer cells was significantly higher than that of normal BEAS-2B cells (Figure 6F). In summary, the difference in CSK activity in cancer cells and non-cancer cells is not obvious, and the relation between the CSK protein and cancer development needs to be further explored.

## 4. Discussion

In this work, we developed a biosensor to monitor the CSK kinase activity in live cells. Using the selected SH2 domain from the previous biosensor, an optimized biosensor containing a piece of the CSK substrate randomly screened from the peptide library was designed. The optimal polypeptide as a substrate is more sensitive in its FRET response to that of a peptide that mimics the tail of the Src C-terminal, and it bears little resemblance to a physiological phosphorylation site. By comparison, in ASM cells, the biosensor showed a clear preference for CSK activity than Src and Fyn kinases.

The CSK-binding protein CBP/PAG-1 not only activates CSK but also recruits CSK into SFK-enriched membrane microregions. This allows CBP to play an important role in the CSK-mediated inactivation of SFKs. Here, we placed the CSK biosensor close to the CBP on the cell membrane via the Fyn, Lyn, and KRas tags to monitor the CSK activity in different membrane microregions. We observed the localization of the Lyn-tag and KRas-tag CSK biosensors by confocal microscopy, while the film localization of the Fyn-tag one was less clear (Figure S3). The subcellular-anchored biosensors showed the spatial resolution required to monitor CSK activity.

CSK kinases are known to be located in these membrane microregions by binding proteins on the cell membrane. Biosensors labeled with the KRas tag had more and stronger FRET responses to PDGF stimuli than sensors labeled with the Fyn and Lyn tags (Figure 1D–F), although the three membrane-targeted biosensors showed similar dynamic ranges in their FRET changes (Figure 1H). Previous studies have shown that CSK is recruited by CBP, which is located at lipid raft regions, and then activated CSK phosphorylates SFKs to result in SFK inhibition [14,15]. A study has also shown that the Src kinase is mainly located at the non-lipid raft regions through N-terminal myristoylation but without N-terminal palmitoylation [42]. In order to keep Src kinase inactive, the Src–CSK complex may remain at the non-lipid raft region. At the same time, CSK may form a complex with other SFK members, such as Fyn and Lyn, at the lipid raft regions. In fact, it has been reported that CSK is located at both raft and non-raft membrane regions in cells, although CBP is mostly located at rafts [53]. Together with our observations based on the FRET measurements, the activity of the CSK kinase exists in both lipid raft and non-lipid raft regions, while it is more dominant in non-lipid raft regions. It will also be interesting to learn more about how these microdomains are classified into different segments based on their specific functional molecules. Therefore, biosensors with specific targeted markers may be useful in characterizing the subtle differences between different membrane microdomains and investigating the underlying mechanisms of local molecular regulation.

PTPα can dephosphorylate pTyr527 of c-Src for activation, while CSK can phosphorylate Tyr527 for inhibition, so we examined the relationship between these two proteins acting on Src. Our observations showed that PTPα had a certain inhibitory effect on the FRET response of the CSK biosensor (Figure 5). Because these two proteins are positive and negative regulators of the Src kinase, respectively, there might be an antagonism between the two. However, how PTPα interacts with CSK remains to be explored.

SFKs, especially Src, can participate in the tumorigenesis process by activating STAT transcription factors [54]. A variety of human tumors have high levels of Src kinase activity expression—for example, breast, colorectal, and pancreatic cancers [55]. The increase in Src kinase activity in the tumor may be caused by tyrosine phosphatase-mediated dephosphorylation, increased Src levels, or upstream regulatory proteins. CSK is a negative regulator of Src, so we further investigated the expression of the CSK kinase in cancer cells. Based on our observations, we found that there was no significant difference in the expression of the CSK kinase in cancer cells and non-cancer cells (Figure 6). As measured by FRET biosensors, CSK’s activity is relatively mild in comparison to the Src and Fyn kinases, which may not offer much variability. Thus, whether there is a correlation between the expression of the two kinases in cancer cells needs to be explored.

The design strategy of the CSK biosensor was similar to that of our previous Fyn biosensor [25], but it measures different kinases’ activity. There are many tyrosine kinases in cells, and their importance underlies their own substrate specificity for different functions. Therefore, any kinase dysfunction would likely cause disorders, including cancer. This specific aspect highlights the need for the development of different biosensors for these kinases in order to study their cellular functions and regulation.

In summary, we have developed a FRET-based CSK biosensor that can detect CSK activity in living cells with a high spatiotemporal resolution. Positioning the CSK biosensor in membrane microregions via acylation and prenylation can further provide a powerful tool for the monitoring of dynamic molecular activity in subcellular compartments. Our results show that CSK activity exists in both the non-lipid and lipid rafts of membranes, while it is more localized in the non-lipid raft regions. Hypothetically, in line with previous studies, after the binding protein CBP/PAG-1 is phosphorylated by SFKs, the CSK kinase is recruited using the phosphotyrosine motif that it contains, and it binds to its SH2 domain, resulting in the CSK kinase being close to the activated Src kinase, phosphorylating the C-terminal tyrosine residue of the Src kinase, inhibiting the Src activity, and maintaining the inactivated Src in the non-lipid raft region, together with the complex formed by the CSK kinase.

## Data Availability

Data are contained within the article and Supplementary Materials.

## Supplementary Materials

The following supporting information can be downloaded at: https://www.mdpi.com/article/10.3390/bios14040206/s1, Figure S1: Molecular sizes of the purified biosensor proteins.; Figure S2: Characterizations of CSK biosensor with different substrate peptides in HeLa and MEF cells. Figure S3: Confocal images of ASM cells show membrane localizations of CSK biosensor with membrane-targeting peptides. Figure S4: FRET change of CSK biosensor activated with PDGF. Movie S1–S4. PDGF-induced FRET responses of cytosolic and different membrane microdomain-localized CSK biosensors in ASM cells. Reference [56] is cited in the Supplementary Materials.
