# Supplementary material for: Development of FRET Biosensor to Characterize CSK Subcellular Regulation
Source: Biosensors (Basel). 2024 Apr 20;14(4):206. doi: 10.3390/bios14040206 (PMC11048185; doi:10.3390/bios14040206)
Supplement: Supplementary file 1 [file biosensors-14-00206-s001.zip › biosensors-2936291-supplementary.pdf]

Supporting Information

Development of FRET Biosensor to Characterize CSK Subcellular Regulation

Mingxing Ouyang [1,*,†], Yujie Xing [1,2,†], Shumin Zhang [1], Liting Li [1,2], Yan Pan [1] and Linhong Deng [1,*]

[1] Institute of Biomedical Engineering and Health Sciences, School of Medical and Health Engineering, Changzhou University, Changzhou 213164, China; xyujie1128@163.com (Y.X.); zsm@cczu.edu.cn (S.Z.); liliting190@163.com (L.L.); py@cczu.edu.cn (Y.P.)
[2] School of Pharmacy, Changzhou University, Changzhou 213164, China
* Correspondence: mxouyang@cczu.edu.cn (M.O.); dlh@cczu.edu.cn (L.D.)
† These authors contributed equally to this work.

The list of supporting materials:

1. Supporting experimental procedures

2. Supporting figures S1-S5

3. Supporting movie

Supporting Experimental Procedures

Cell culture

HeLa cells were cultured in high-glucose DMEM (Gibco) supplemented with 10% fetal bovine serum (FBS), 2 mM L-Glutamine, 100 I.U./ml penicillin, and 100 μg/ml streptomycin and maintained at 37°C in a 5% CO2 incubator.

Purification of biosensor and molecular size with Coomassie brilliant blue staining

The recombinant biosensor proteins with an N-terminal 6xHis tag in the pRSETb vector were expressed in E. coli (BL21) by culturing with 0.4 mM isopropyl β-D-1-thiogalactopyranoside (IPTG, Sigma-Aldrich , St. Louis, MO, USA) at 25°C for 16-24 hours and purified by nickel chelation chromatography[1].

The purified protein was subjected to 10% sodium dodecyl sulfate polyacrylamide gel electrophoresis (SDS-PAGE) to separate the proteins based on their sizes. Following electrophoresis, the gel was washed with double-distilled water using agitation. Subsequently, the gel was immersed in a Coomassie brilliant blue staining solution and agitated for 30 minutes to facilitate the binding of the dye with the proteins. The gel was then incubated in the destaining soultion (containing 50% ethanol and 10% acetic acid) through multiple washes to remove the unbound dye. Images of the protein bands in the gel were recorded by a digital camera.

Supporting Figures

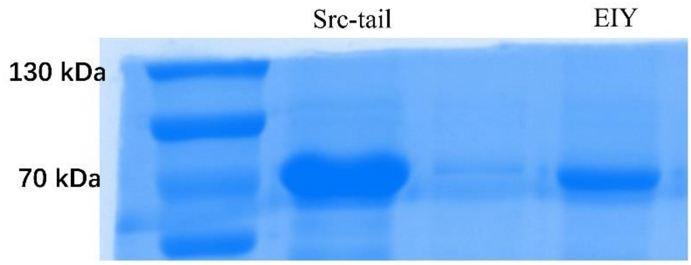

Figure S1. Molecular sizes of the purified biosensor proteins. The biosensor proteins were resolved on an SDS-PAGE gel by electrophoresis and stained with Coomassie bright blue. The picture shows the successfully purified CSK biosensor proteins with Src-tail and EIY substrates, displaying a molecular size of around 70 kDa.

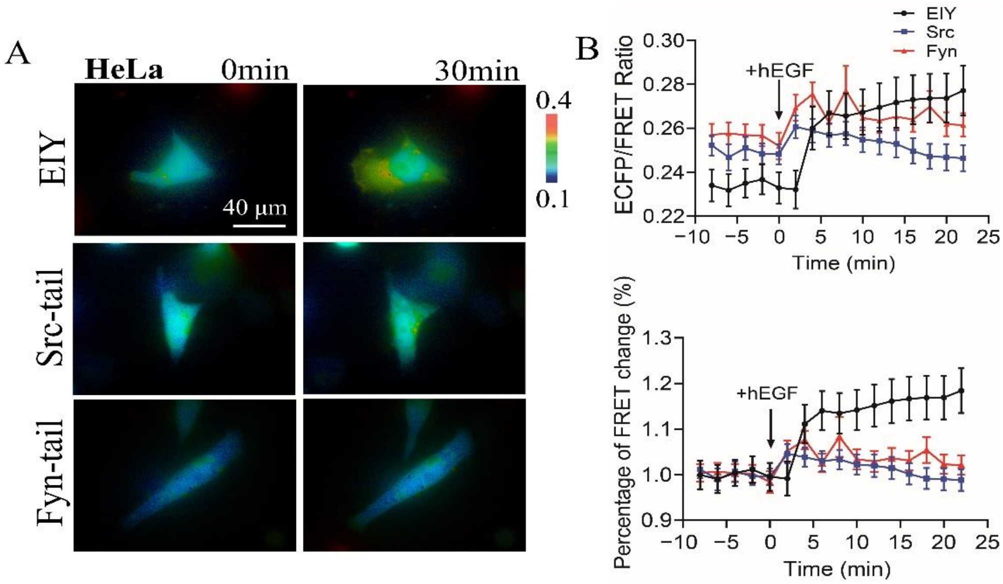

Figure S2. Characterization of CSK biosensor with different substrate peptides in HeLa and MEF cells. (A) The ratiometric FRET images of HeLa cells expressing the CSK biosensor with the EIY, Src-tail, or Fyn-tail substrate before and after EGF stimulation. **(B)** The quantified time courses of the FRET ratio and normalized FRET changes from the cells in (A).

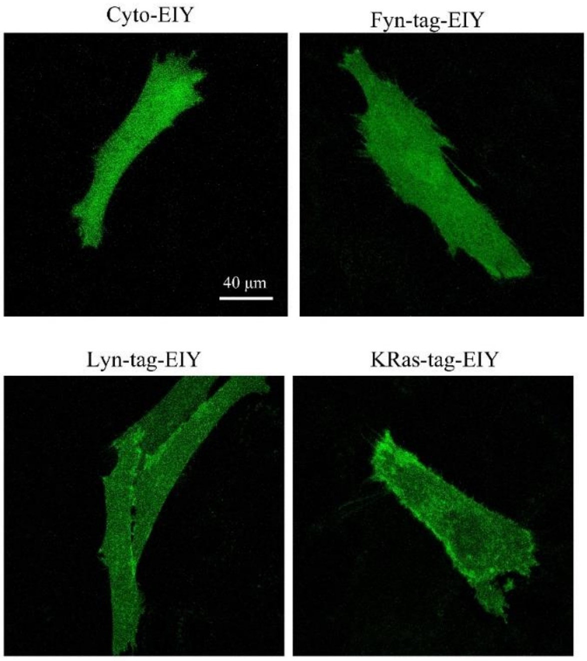

Figure S3. Confocal images of ASM cells show membrane localization of CSK biosensor with or without membrane-targeting peptides. ASM cells were transfected with cytosolic or membrane microdomain-targeted CSK biosensors (Cyto, Fyn-tag, Lyn-tag, KRas-tag). After 24 h, cells were further seeded onto glass-bottom dishes overnight for confocal microscopic imaging.

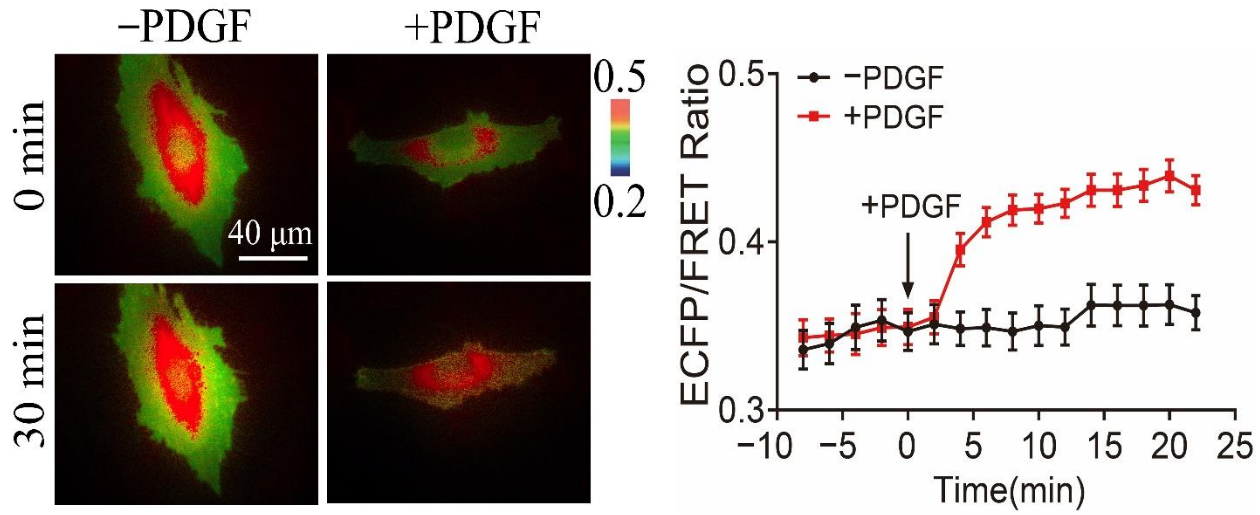

Figure S4. FRET change of CSK biosensor activated with PDGF. The KRas-tag-

CSK FRET biosensor was transfected into ASM cells, followed by FRET imaging without (control group) or with (experimental group) 10 μg/ml PDGF stimulation. The changes in the FRET ratio were compared through the quantified time courses with or without PDGF.

Supporting movie caption

Supporting Movie S1-S4. PDGF-induced FRET responses of cytosolic and different membrane microdomain-localized CSK biosensors in ASM cells.

Reference:

1. Miyawaki, A.; Tsien, R. Y., Monitoring protein conformations and interactions by fluorescence resonance energy transfer between mutants of green fluorescent protein. *Methods in enzymology* **2000,** *327*, 472-500.
